# Evaluation of Individual and Crystal Population Dissolution Rates by Time-Resolved X-ray Microtomography

**DOI:** 10.1021/acs.cgd.4c00113

**Published:** 2024-04-03

**Authors:** Filip Hládek, David Zůza, Ondřej Navrátil, Jan Tomas, Aleš Zadražil, Vladimír Novák, František Štěpánek

**Affiliations:** † Department of Chemical Engineering, University of Chemistry and Technology, Prague, Technická 5, Praha 166 28, Czech Republic; ‡ Zentiva, k.s., U Kabelovny 130, Praha 10 102 00, Czech Republic; § Paul Scherrer Institute, Swiss Light Source, Forschungsstrasse 111, Villigen PSI 5232, Switzerland

## Abstract

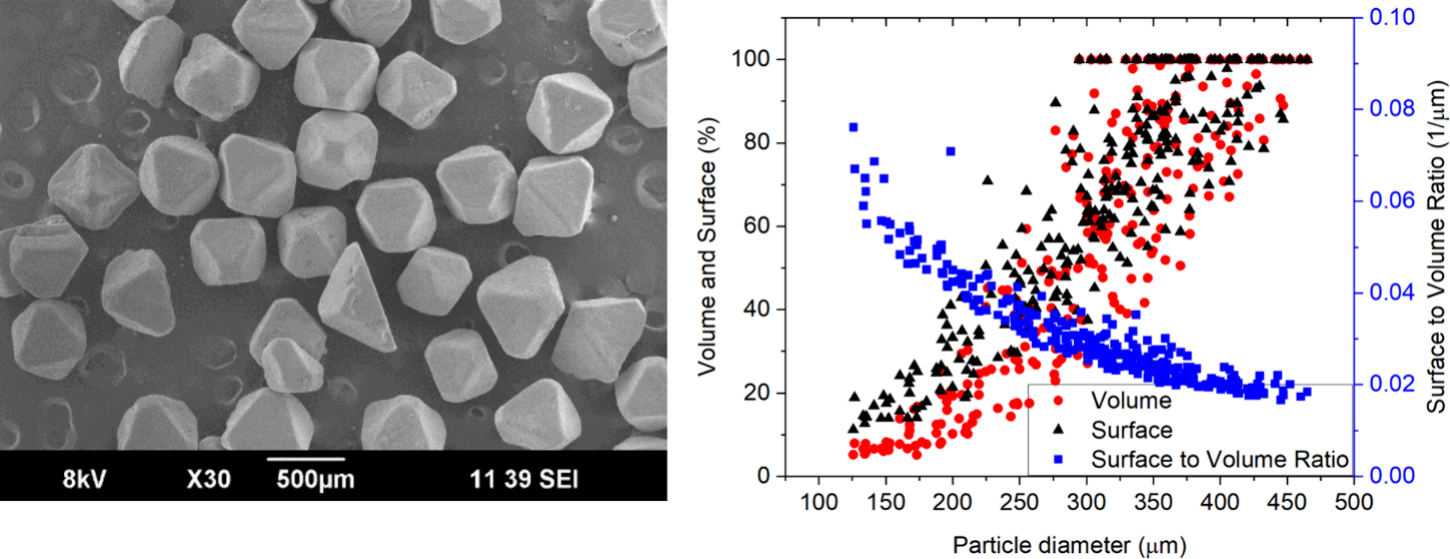

The intrinsic dissolution rate (IDR) is an important parameter in pharmaceutical science that measures the rate at which a pure crystalline active pharmaceutical ingredient dissolves in the absence of diffusion limitations. Traditional IDR measurement techniques do not capture the complex interplay between particle morphology, fluid flow, and dissolution dynamics. The dissolution rate of individual particles can differ from the population average because of factors such as particle size, surface roughness, or exposure of individual crystal facets to the dissolution medium. The aim of this work was to apply time-resolved X-ray microtomography imaging and simultaneously measure the individual dissolution characteristics of a large population of crystalline particles placed in a packed bed perfused by the dissolution medium. Using NaCl crystals in three different size fractions as a model, time-resolved microtomography made it possible to visualize the dissolution process in a custom-built flow cell. Subsequent 3D image analysis was used to evaluate changes in the shape, size, and surface area of individual particles by tracking them as they are dissolved. Information about the particle population statistics and intrabatch variability provided a deeper insight into the dissolution process that can complement established IDR measurements.

## Abstract 2

The dissolution kinetics of a population of crystals has been captured by time-resolved tomography imaging. Using an advanced segmentation and tracking algorithm, the shape and size evolution of each crystal in the population was evaluated, and the distribution of the individual dissolution rates within the population was compared with the intrinsic dissolution date measure in bulk, providing crucial insight into intrabatch variation of particle properties.

## 1 Introduction

The intrinsic dissolution rate (IDR) is a crucial parameter in pharmaceutical sciences as it can guide formulation and solid-state development activities.^1^ A low IDR may indicate insufficient dissolution of the active pharmaceutical ingredient when administered as a solid dosage form and subsequently low oral bioavailability. In a standardized setup, IDR is measured by compressing the tested powder sample into a compact disk, which is then exposed to the dissolution medium under intense stirring.^2^ The IDR is evaluated from the concentration change of the dissolved substance and is usually expressed in units of mass dissolved per unit area per unit time. Although the IDR is relatively easy to measure and provides a useful common basis for comparing different drug substances, it also has limitations. One limitation is the assumption of a constant surface area of the solid material during dissolution, which fails to account for the heterogeneity in particle dissolution rates arising from variations in size, morphology, and crystal facet exposure.^3^ Another limitation is averaging.^4^ It is well-known that the dissolution rate from individual facets or crystal planes may not be identical.^5,6^ Thus, the same substance may yield different IDR values due to the different proportions between individual crystal facets in the overall surface area of the sample exposed to the dissolution medium. This may be the consequence of a previous milling step in which crystal cleavage occurs preferentially in certain fracture planes.^7,8^ Such nuances are particularly important for poorly water-soluble active pharmaceutical ingredients, where even small changes in the IDR can significantly affect the oral bioavailability and therapeutic efficacy.^9^ Thus, a method that could capture the shape evolution of many particles during dissolution simultaneously with conventionally unreachable spatial and temporal resolution would be beneficial.

To capture particle morphology changes and observe the dissolution of individual particles, various imaging approaches have been reported in the literature. For example, 3D X-ray microtomography has been used^10,34,35^ as well as nonlinear optical imaging or real-time ultraviolet imaging.^11^ Multiple studies have explored the possibilities of utilizing microtomography for otherwise impossible nondestructive in-depth view of solid dosage forms, such as pharmaceutical tablets.^12,13^ X-ray microtomography has also been used for the purpose of visualization of API release from a solid dosage form.^14^ These studies grant valuable insights into material properties formed during the solid dosage form preparation and use.^15^

Imaging methods show great promise in the field of single-crystal dissolution description and can help explain various phenomena around crystalline formulations and their behavior. However, they are generally not capable of describing collective properties of crystal populations with a polydisperse size or shape distribution. To obtain statistically meaningful results, at least several tens or hundreds of individual crystals should be analyzed, which would be very time-consuming in the case of one-by-one observation. An ideal particle dissolution imaging method should be fast, allow for the simultaneous observation of many individual crystals, and do so in 3D such that all possible crystal orientations relative to the flow direction of the dissolution medium can be statistically represented. Laboratory-based X-ray computed tomography (XRT) allows for investigation in 3D pharmaceutical dosage forms including the distribution of different ingredients and their internal structure.^16,17^ The XRT has been also used to visualize the dynamic behavior of the pharmaceutical dosage forms during the dissolution process.^18^ As this method is based on X-ray attenuation contrast, which depends on the atomic number and density of the material, it is thus often impossible to distinguish weakly absorbing pharmaceutical materials from the dissolution medium. To overcome this limitation, contrast agents in the form of soluble salts, such as CaI or KI, can be added to the dissolution medium to increase the absorption contrast. With the development of synchrotron sources and the availability of the partially coherent beam, the visualization of weakly absorbing materials has become possible.^19^ The so-called synchrotron X-ray phase-contrast computed microtomography (SR-pXRT) provides information about the refractive index of a material, in addition to its attenuation coefficient. The phase contrast modality takes advantage of a stronger refractive effect compared to the attenuation.^20^ In addition, the high intensity of synchrotron radiation enables tomographic scanning with subsecond acquisition speed.^21^

In this work, we used the SR-pXRT to follow in 4D (3D + time) the dissolution of NaCl crystals. An experimental setup was built that contains a mixture of insoluble Al_2_O_3_ and soluble NaCl particles in a packed bed perfused by the dissolution medium. The insoluble particles function as spacers that allow undisturbed observation of NaCl dissolution. A 3D particle tracking algorithm was developed to automatically segment and evaluate morphological parameters such as volume, surface area, and shape descriptors of each particle in a tested population at discrete time intervals. Thus, both single-crystal and population-level dissolution phenomena could be analyzed, providing information unattainable by classical IDR measurement.

## 2 Materials and Methods

### 2.1 Chemicals

Sodium chloride (NaCl) was selected as a soluble model substance with high visibility on X-ray and aluminum oxide (Al_2_O_3_) particles were selected as an insoluble matrix. Both materials were purchased from Sigma-Aldrich. Isopropyl alcohol (IPA, > 98%) was purchased from Penta. Deionized water (Aqual 25, 0.07 μS·cm^–1^) was used for all reactions and treatment processes.

### 2.2 Particle Processing and Analysis

The alumina particles (Al_2_O_3_) that functioned as inert spacers were sieved, and a fraction of 100–140 μm was used in all experiments. NaCl, which was used as a model material for dissolution experiments, was sieved into the following fractions: 180–250 μm (further denoted as small – S), 300–400 μm (denoted as medium - M) and 400–500 μm (denoted as large – L). The complete particle size distribution of each sieve fraction was measured by dynamic image analysis using the Microtrac CAMSIZER X2 with isopropyl alcohol as a dispersion medium in which NaCl is insoluble. The morphology of the NaCl crystal particles was analyzed by optical microscopy (Olympus BX41) and scanning electron microscopy (Jeol JCM-5700 SEM).

### 2.3 Dissolution Media

To achieve dissolution rates typical of water-soluble pharmaceutical substances using NaCl as a model material, the dissolution medium was created by mixing isopropanol and water in a volumetric ratio of 2:1. Available data for phase equilibria in a ternary system comprised of water, isopropyl alcohol and NaCl show a significant decrease in solubility of NaCl as the fraction of isopropyl alcohol in the system rises.^22^ The addition of isopropyl alcohol also slightly reduces the dissolution rate while still allowing the dissolution of NaCl crystals to take place.^36^

### 2.4 Experimental Setup

**Figure 1:**
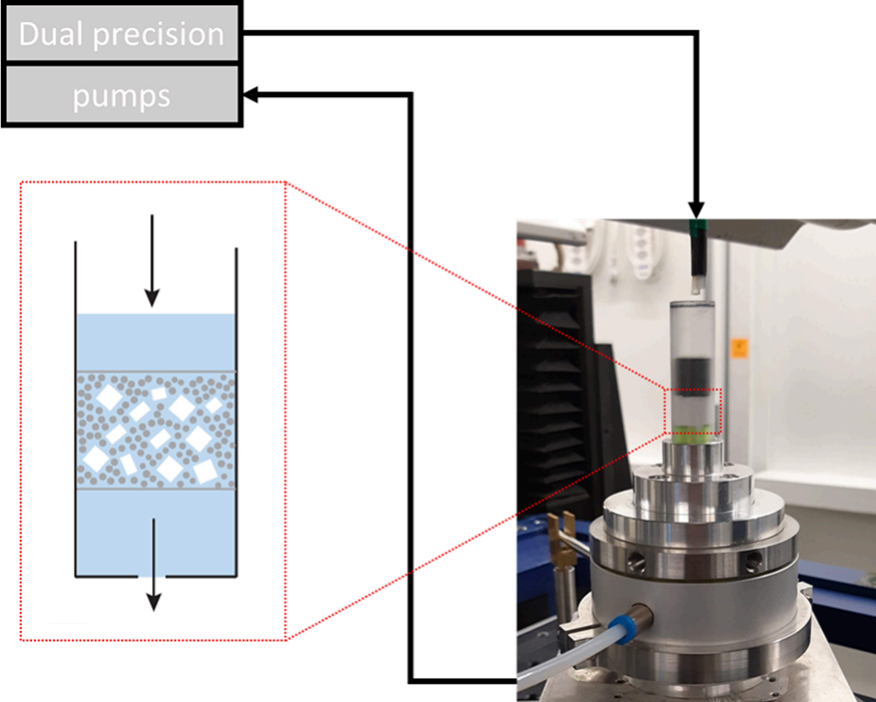
Scheme of the experimental setup with a continuous flow of dissolution medium through a rotating sample holder designed for synchrotron X-ray microtomography. The diagram represents the detail of the packed bed containing inert alumina particles surrounding distributed NaCl crystals.

A custom-built sample holder for imaging of NaCl dissolution was made from a polycarbonate tube (d = 1 cm) to minimize the X-ray absorption (Figure 1). NaCl crystals (15% w/w) were uniformly dispersed and immobilized in a randomly packed bed of alumina particles filled into the polycarbonate tube, kept in position by an 80 μm wire mesh supporting the bottom and a 35 μm wire mesh covering the top. The sample holder was placed into an in-house modified rotary union (JR 1–1–4 R40 from TDS Precision Products GmbH). At the beginning of the experiment, the tube was filled with the dissolution medium from a tube suspended above the sample holder, as shown in Figure 1. The volumetric flow rate during filling of the sample holder was 10 mL·min^–1^. Once filled, the pumps were switched into the continuous flow of the dissolution medium through the sample holder with a volumetric flow rate of 1 mL·min^–1^. The continuous flow was achieved by constant refilling from the top and simultaneous suction from the bottom. The suction tube was connected through the rotary union to allow continuous rotation of the setup, which is mandatory for tomographic imaging. The liquid delivery was done by using two linear pumps (CETONI, Nemesys).

### 2.5 Time-Resolved X-ray Microtomography of Crystal Dissolution

The SR-pXRT was conducted using the TOMCAT beamline (Swiss Light Source). Filtered polychromatic X-ray radiation with a peak energy of approximately 26 keV, originating from a 2.9 T bending magnet source, was used for all experiments. A high-resolution white-beam microscope (Optique Peter) with 4× magnification was combined with the in-house developed GigaFRoST high-speed camera.^23^ The effective pixel size was 2.75 μm, and the field-of-view was 2016 × 2016 pixels, resulting in a scanned section size of about 5.544 × 5.544 × 5.544 mm^3^. A total of 1000 projections were acquired per scan, with an exposure time of 1 ms per frame. Consequently, the total time to acquire a single scan was 1 s, and subsequent scans were acquired every 6 s to reduce the amount of the collected time-series data. During the 5 s pause between two scans, a fast X-ray shutter was closed to prevent unnecessary sample exposure to the beam.

### 2.6 Image Reconstruction, Processing, and Data Analysis

**Figure 2:**
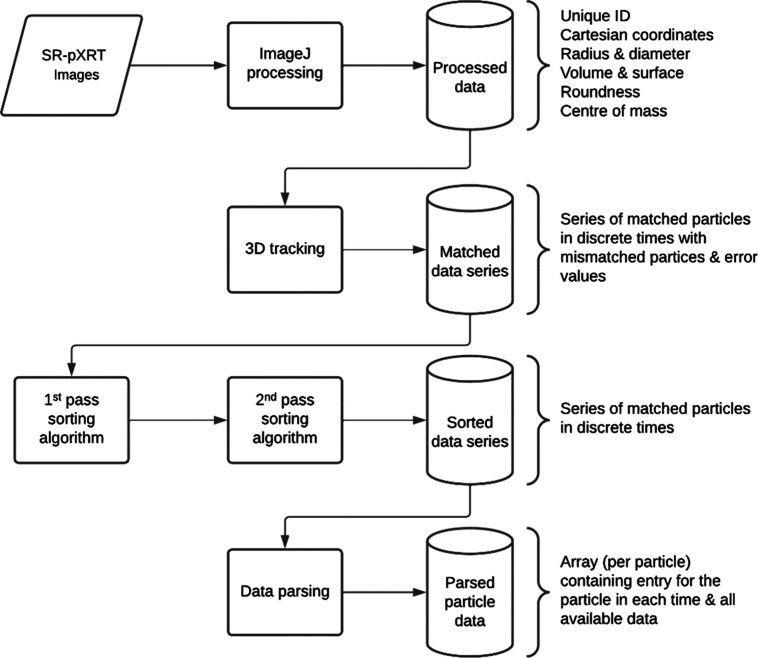
Data analysis workflow is represented as a block diagram.

The 3D volume data were reconstructed using the propagation-based phase contrast method^24^ and the Gridrec algorithm.^25^ The acquired stack of images was processed using ImageJ/Fiji.^26^ The evaluated area was cropped to a 2016-pixel diameter circle in the *X*–*Y* plane to exclude the sample from the reconstruction circle. Subsequently, the alumina particles were segmented out by gray value thresholding. The remaining NaCl crystals were then evaluated as 3D objects to acquire data for the 3D particle surface tracking and subsequent analyses. The Li thresholding algorithm,^27^ followed by binarization was used to separate NaCl particles from the background. To obtain the necessary data, the 3D object counter plug-in was used. ImageJ preprocessing generated a data entry for each crystal measured at each time point of the dissolution experiment (at time = 0 and then every 6 s): a unique ID was assigned to each crystal, and its Cartesian coordinates, radius, diameter, surface, volume, roundness, and center of mass were calculated at each time point (Figure 2, Processed data), resulting in a database of all uniquely described crystals. All subsequent calculations were based on these values.

As crystals may shift in position or even break up into several particles during their dissolution within the packed bed, it was crucial to properly track their identity and calculate the dissolution rate of the same crystal by following its volume changes between time points. The crystals in the data series were matched by calculating the tracking parameter for each crystal (with a unique ID) at each time point, which was defined as the smallest Euclidean distance between the coordinates of the crystal center of mass at time *t* + 1 and the coordinates of any crystal at time *t* (Figure 2, 3D tracking). This tracking system makes it possible to distinguish between situations where crystal A at coordinates *X*, *Y*, *Z* at time *t* moved to a new set of coordinates, *X* + d*X*, *Y* + d*Y*, *Z* + d*Z* at time *t* + 1, while crystal B in the time step *t* + 1 moves to the previous coordinates of crystal A at *X*, *Y*, *Z*.

While this process successfully identifies the correct sequence of crystals throughout the measurement period, it may lead to nonunique crystal ID assignment in the case of crystal breakup. Nonuniquely identified crystals are then paired to their mother particle, and redundant ID assignments are removed by an in-house developed sorting algorithm. This algorithm was applied in two passes: during the first pass, some nonuniquely identified crystals were removed based on the premise that crystals cannot grow during dissolution (based on a comparison of surface and volume development in time). During the second pass, the algorithm looked at unusual or improbable sudden changes in morphological descriptors between time points, further removing nonuniquely identified crystal series.

The crystals remaining in the data set after applying the filters were then matched together in the longest possible sequence on a series of parameters (e.g., volume, surface area, etc. as functions of time). The absolute value of the sum of coordinate deviation and volume decrease caused by the dissolution was calculated, and crystals with the smallest coordinate shift with an acceptable volume decrease (crystals with a clear decrease that did not dissolve completely at the initial times) were favored in the sequence. Filtering resulted in a data series for each crystal that contained a single data entry for each time (Figure 2, Sorted data series). The selection of data was quality-checked by comparing the algorithm-produced data series with visual observation of the measured data, confirming that the filters worked correctly.

The individual dissolution rate of each crystal was then evaluated according to the formula:
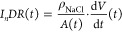
1where *I*_*n*_*DR*(*t*) is the individual crystal dissolution rate in mg·cm^–2^·min^–1^, ρ_*NaCl*_ is the material density in mg·cm^–3^, *A*(*t*) is the crystal surface area in cm^2^, and  is the crystal volume change rate in cm^3^·min^–1^. For evaluation of the derivative in eq 1, the volume and surface at discrete time steps (Figure 2, Parsed crystal data) were regressed by a smooth function (polynomial of the second degree), which was then differentiated analytically. For visual evaluation, the images were processed by ImageJ (MorphoLibJ morphological Open function, focusing on disk, kernel size 15, and connect-6 algorithm^28^) and rendered by DragonFly.^29^

The I_n_DRs of all crystals were then analyzed statistically to evaluate the population average, the evolution of I_n_DR on time, its dependence on the NaCl particle size class, and also on the particle position within the packed bed both vertically and radially.

### 2.7 Standard IDR Measurement

A standardized intrinsic dissolution rate (IDR) measurement with a rotating disk assembly (the so-called Woods apparatus according to Ph. Eur. 2.9.29^30^) was carried out to acquire comparable data as this method is commonly used in pharmaceutical research. Three NaCl size fractions were used for this test. The fractions were selected from the outermost values of 25–100 μm and 400–500 μm for the most variability and the fraction of 300–400 μm as the middle value. 500 mg of crystals were pressed using a Carver 4350L manual tablet press in Woods apparatus dies (*d* = 0.8 cm with approximately 0.5 cm^2^ area) with a force of 9.81 kN and allowed to yield for 4 min under such pressure.^31^ Each fraction was evaluated in triplicate. Dissolution was conducted on the Vankel Varian VK 7000 USP II apparatus with a round-bottom vessel containing 900 mL of a dissolution medium at a temperature of 24 °C and the dies rotated at the rate of 250 rpm. The concentration of dissolved NaCl was evaluated from online conductivity measurement using the Mettler Toledo SG3 conductivity meter with the probe inserted into the dissolution vessel for which a 5-point calibration curve was measured.^32^

## 3 Results and Discussion

### 3.1 Premeasurement NaCl Crystal Characterization

**Figure 3:**
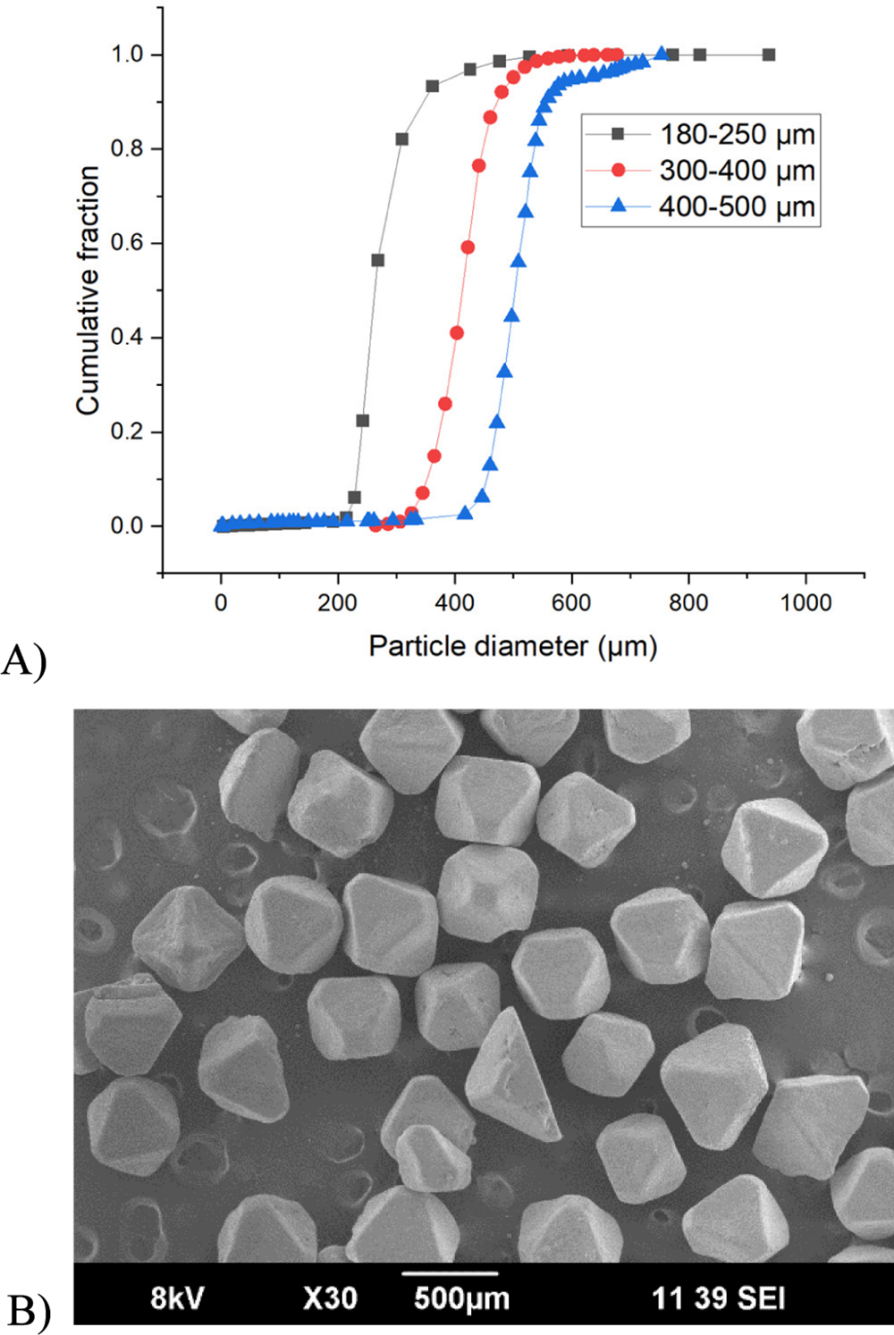
(A) Volume-weighted cumulative particle size distribution of NaCl sieved fractions; (B) representative image of NaCl crystals from SEM.

The particle size distribution of the sieved crystal fractions was measured by a Microtrac CAMSIZER X2. The measurement confirmed a fine division between fractions as expected (Figure 3A). Crystals were also qualitatively analyzed by SEM (Figure 3B). The crystal morphology can be described as smooth with irregularities. The two most frequently observed shapes are an octahedron and a pyramid (half of the octahedron).

### 3.2 Single Crystal Dissolution

**Figure 4:**
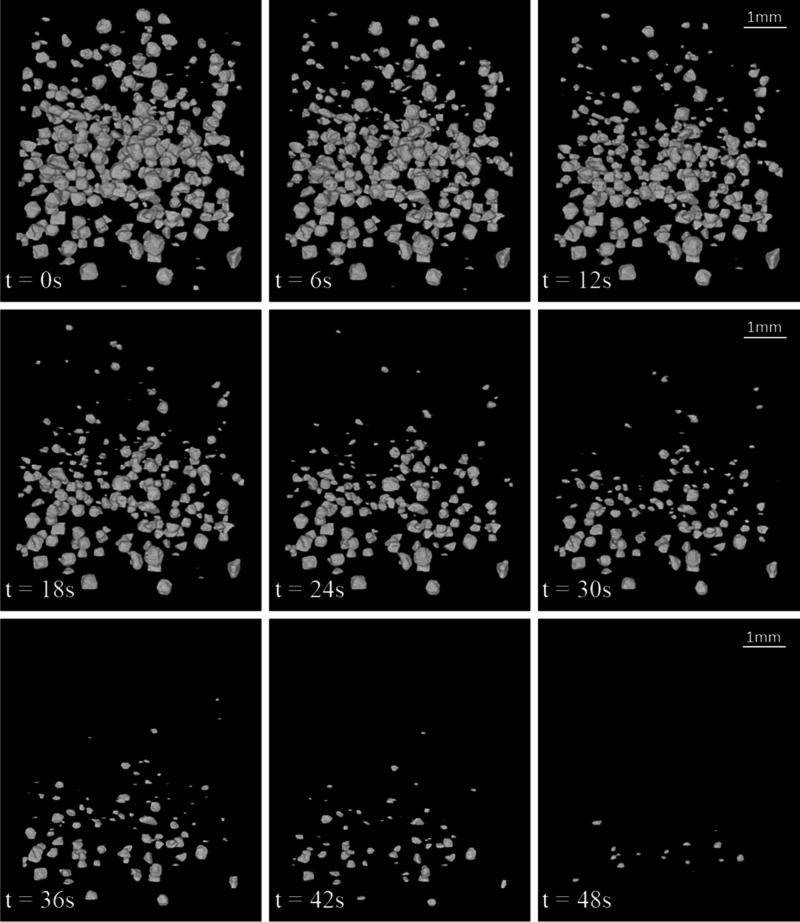
Visualization of one measurement from SR-pXRT at times ranging from 0 s (first image) to 48 s (last image) at 6 s intervals. The scale bar represents 1 mm, the field of view is 5.544 × 5.544 mm.

The acquired data were processed and analyzed as described in the protocol in Section 2.6. Direct visualization of raw data measured by time-resolved microtomography shown in Figure 4 shows how crystals at the top of the stack dissolve first and crystals at its bottom dissolve later. This is because the dissolution medium saturates at the top, meaning the crystals at the bottom are initially washed only by saturated media and cannot dissolve.

Using an automatic tracking and filtering algorithm described in Section 2.6, the crystals were successfully tracked and parsed to a database of individual crystals. The data acquired from the 3D object counter for each crystal at each observed time were the volume, surface area, and spatial position. For each measured data stack, 10–15 crystals were manually cross-checked with data visualization to prove the algorithm’s accuracy. Only crystals appearing in at least 4 subsequent scans (i.e., at least 4 time points) were included in the analyses to enable accurate evaluation of InDR from eq 1. In total, 182 crystals were analyzed, with the least number of crystals analyzed from the smallest size fraction of crystals as their dissolution was too fast to go through the filtering and matching algorithm. The details of the number of crystals analyzed in each size class are summarized in Table 1.

**Table 1:** Summary of Data Acquired for Analysis

**initial crystal size class (μm)**	**successfully tracked crystals**	**data points^a^**	**data points per crystal**
180–250 (S)	22	121	5.5
300–400 (M)	106	587	5.5
400–500 (L)	64	379	5.9

a Data point refers to the number of crystals tracked in each scan for a given size fraction after a successful pass of our pairing algorithm. Only crystals with 4 or more records in at least 4 unique scans in one experiment were included

**Figure 5:**
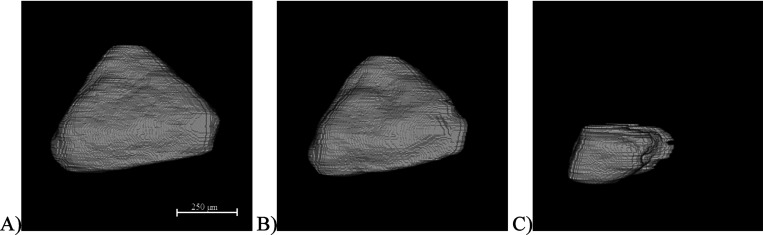
Example of a representative pyramid-shaped crystal dissolution (A) *t* = 0 s, (B) *t* = 12 s, and (C) *t* = 24 s; the scale bar represents 250 μm.

**Figure 6:**
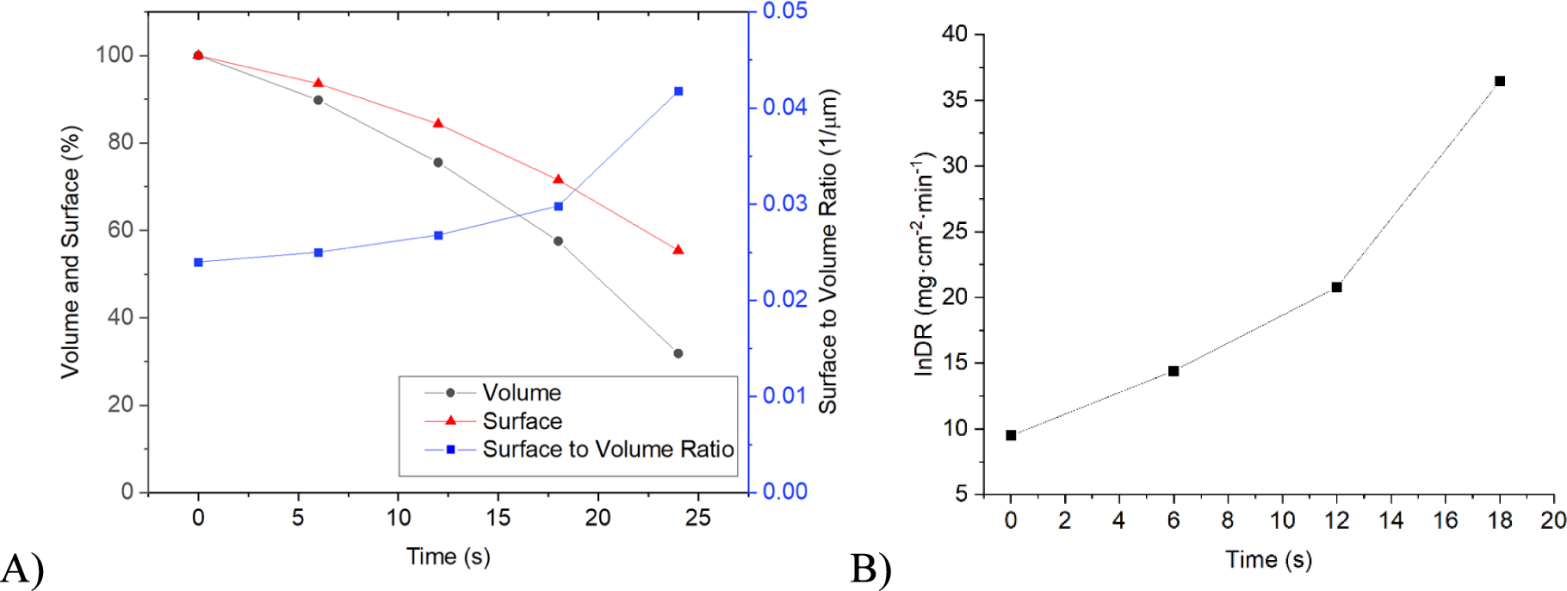
(A) Measured lifetime of the pyramid-shaped crystal from Figure 5 is described by surface, volume, and surface-to-volume ratio, and (B) evolution of individual dissolution rate for the pyramidal crystal from Figure 5 over its tracked lifetime.

All tracked crystals were subjected to shape analysis, which was verified by hand. It was found that for NaCl crystals, the shape is irrelevant for dissolution, i.e., the surface dissolution rate from pyramids and octahedra was not statistically different. A typical representative crystal of a pyramid shape and a typical representative crystal of an octahedral shape were picked as examples of individual crystal tracking. The pyramidal crystal was tracked 5 times by the algorithm. From the SR-pXRT, it can be observed that the shape of the crystal remains the same for most of the process, only changing to a disk-shaped crystal in the final stages of dissolution as the top is washed away (Figure 5). Other base data acquired for the crystal are summarized in Figure 6A. The calculated individual dissolution rate for this crystal increases with time as the crystal gets smaller (Figure 6B).

**Figure 7:**
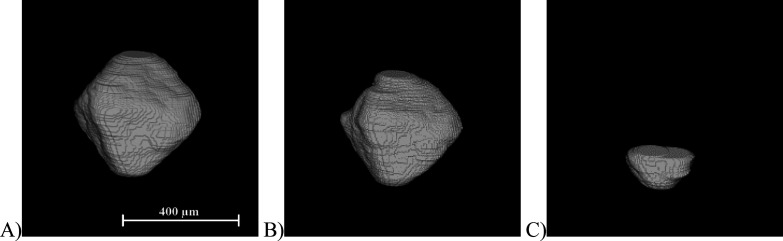
Example of a representative octahedral-shaped crystal dissolution (A) *t* = 0 s, (B) *t* = 24 s, and (C) *t* = 36 s; the scale bar represents 400 μm.

**Figure 8:**
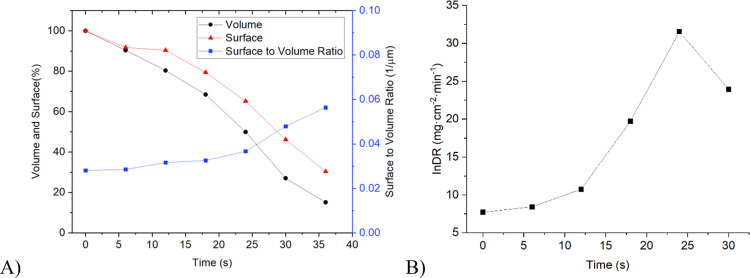
(A) Measured lifetime of the octahedral-shaped crystal from Figure 7 is described by surface, volume, and surface-to-volume ratio and (B) evolution of the individual dissolution rate for the octahedral crystal from Figure 7 over its tracked lifetime.

In the case of the dissolution of an octahedron-shaped crystal (Figure 7), similar trends can be observed. The crystal retains its original shape as the material is washed away from the planes and vertices, similarly decreasing the overall size. In later dissolution stages, sharp edges and peaks are washed away faster, causing a transfer from an octahedron to a disk-like shape. Data acquired throughout the lifetime are summarized in Figure 8. The individual dissolution rate for octahedron-shaped crystals increases with the lifetime of the crystal, but at the end, it oscillates a bit back, which is common behavior observed nonspecifically throughout the experiments. This decrease in the dissolution speed may be the result of a local increase in the saturation of the solution.

### 3.3 Dissolution of Crystal Population

**Figure 9:**
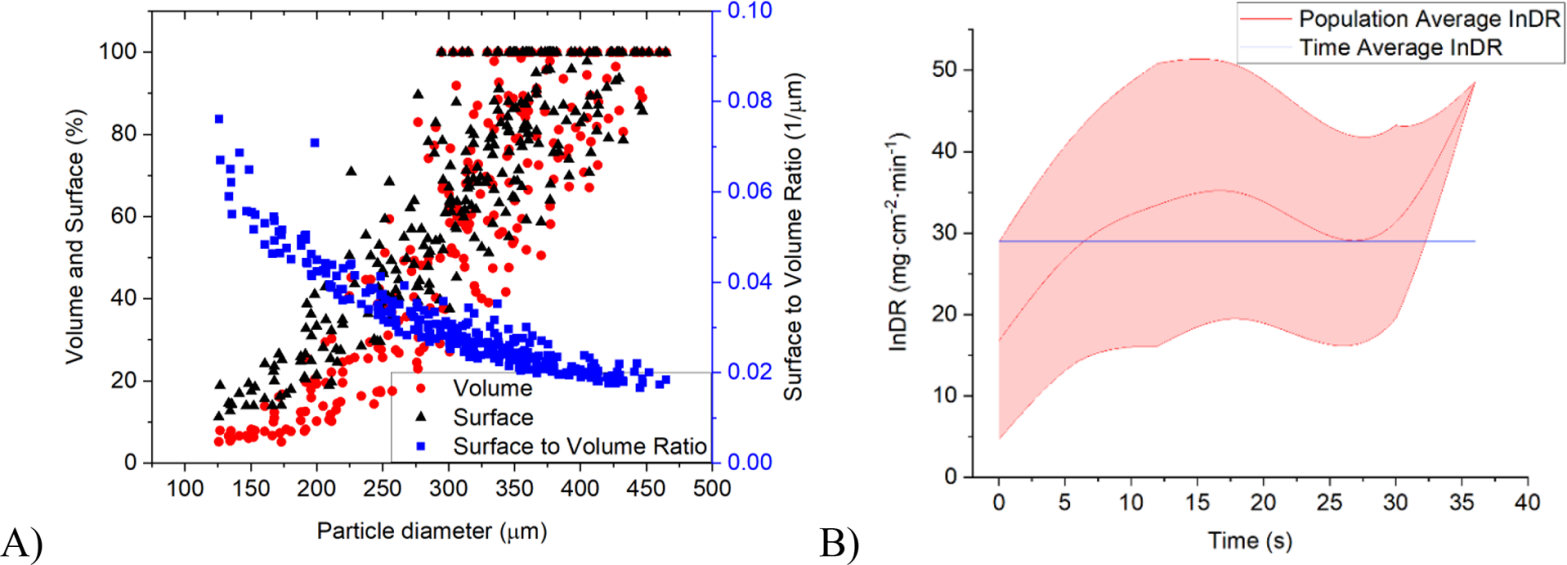
(A) Population data for volume, surface, and surface-to-volume ratio evaluated from dissolution experiments for crystals from the L (400–500 μm) size class; (B) time-average individual dissolution rate compared to the population-average as a function of time for the *L* size class of crystals. The purple region denotes the range of individual values.

**Figure 10:**
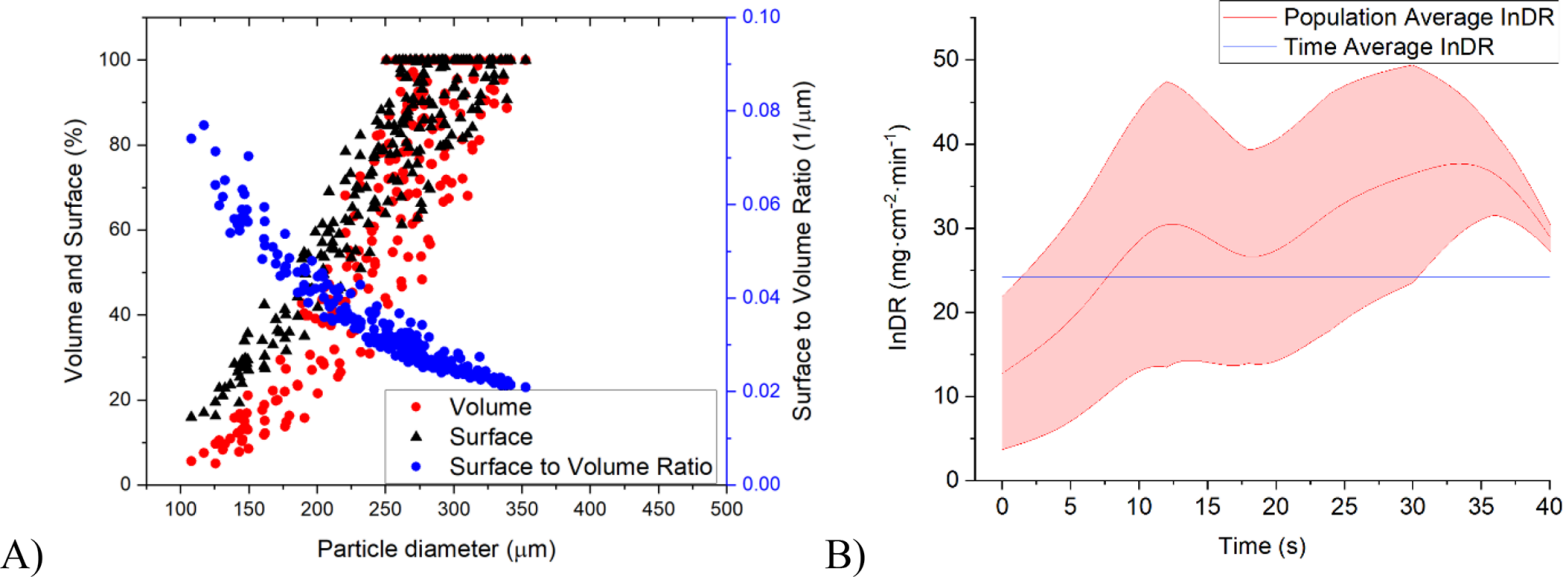
(A) Population data for volume, surface, and surface-to-volume ratio evaluated from dissolution experiments for crystals from the M (300–400 μm) size class; (B) time-average individual dissolution rate compared to the population-average as a function of time for the M size class of crystals. The purple region denotes the range of individual values.

**Figure 11:**
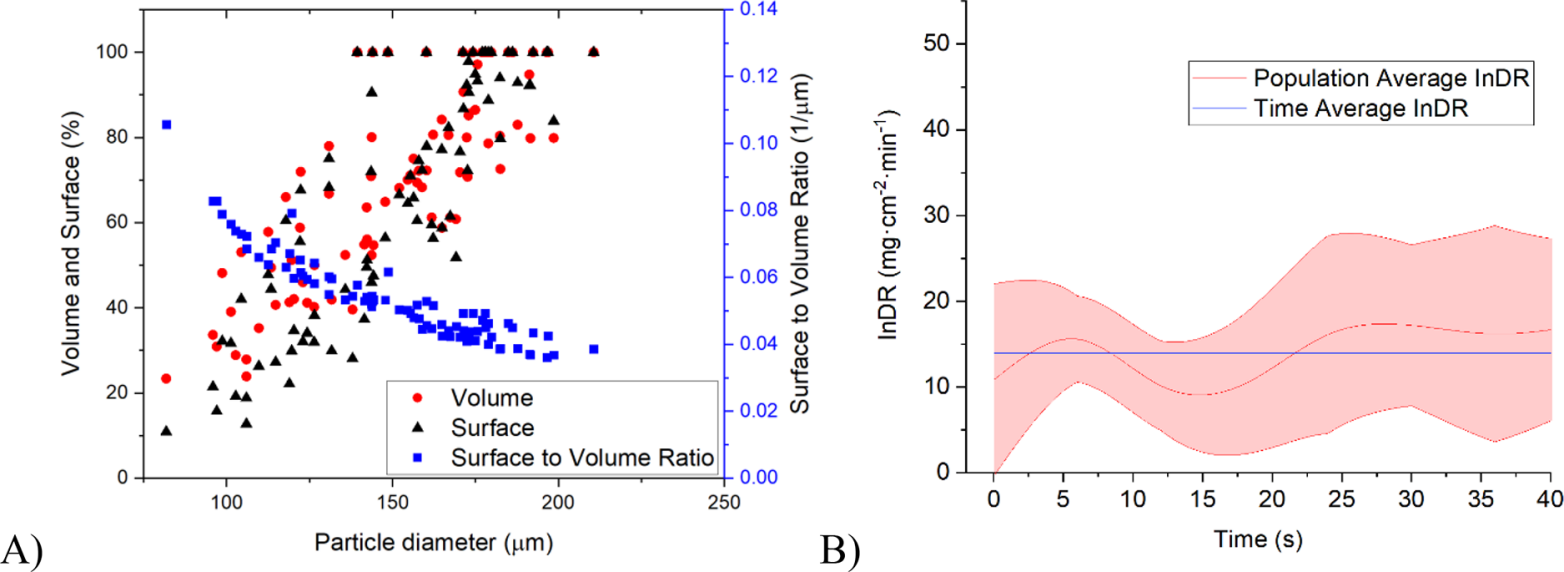
(A) Population data for volume, surface, and surface-to-volume ratio evaluated from dissolution experiments for crystals from the S (180–250 μm) size class; (B) time-average individual dissolution rate compared to the population-average as a function of time for the *S* size class of crystals. The purple region denotes the range of individual values.

The same analysis that was shown in the previous section for two representative crystals (Figures 5–8) was then repeated for all 182 crystals identified by the algorithm. In each time step, the average crystal dissolution rate of each size class (S, M, L) was then evaluated from the individual dissolution rates (I_n_DR) of all crystals that were tracked. The crystal lifetime data used for the calculation are shown in Figures 9A, 10A, and 11A for the L, M, and S size classes, respectively. The mean I_n_DR value for the large size class was 29.03 mg·cm^–2^·min^–1^, for the medium size class 24.20 mg·cm^–2^·min^–1^, and for the small size class 13.73 mg·cm^–2^·min^–1^. The averages and ranges for each size fraction are depicted in Figures 9B–11B, and they indicate a relatively broad variation within the crystal population. Such intrapopulation variation would not be captured by bulk dissolution methods and highlights the advantages of time-resolved microtomography as a method of particle dissolution measurement. The decrease in the dissolution rate for smaller crystals is probably caused by the low number of crystals that were not excluded by the algorithm and the higher likelihood that those crystals are not receiving enough fresh dissolution media.

### 3.4 Comparison of Standard IDR Measurement with SR-pXRT

The standard intrinsic dissolution rate (IDR) measurement was conducted by the disc method as described in Chapter 2.7. For large crystals, the calculated value of IDR was 26.60 mg·cm^–2^·min^–1^, for medium-sized crystals 26.97 mg·cm^–2^·min^–1^, and for extra small crystals 26.89 mg·cm^–2^·min^–1^. The confidence interval for the linear regression was calculated with confidence level α = 0.95. All IDR values do fall within the confidence intervals of each crystal size class. This result shows how robust standard methodology is (Table 2) and implies that the uniformly pressed surface of the material in the die can strongly diminish otherwise observable variance in the dissolution rate for differently sized crystals.

**Table 2:** Standard Intrinsic Dissolution Rate Measured by the Disc Apparatus

**crystal size class** (μm)	**dissolution surface** (cm^2^)	**IDR** (mg·cm^–2^·min^–1^)	**95% confidence interval** (mg·cm^–2^·min^–1^)
25–100 (S)	0.5027	26.89	(26.28; 27.50)
300–400 (M)	0.5027	26.97	(26.45; 27.48)
400–500 (L)	0.5027	26.60	(26.04; 27.15)

**Figure 12:**
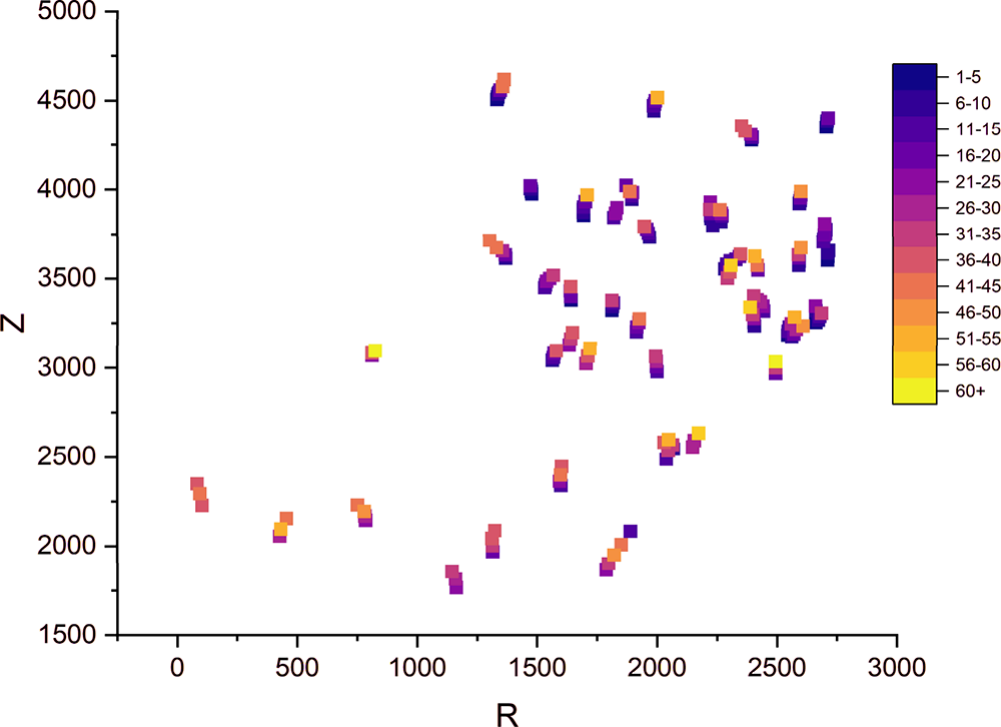
Distribution of I_n_DR for L class crystals (R – radial coordinate from the cell axis measured in voxels, Z – depth coordinate measured in voxels, Z = 0 represents the upper boundary of the measurement cell shown in Figure 1).

Direct comparison of bulk averaged I_n_DR with IDR (Table 3) shows that values evaluated from SR-pXRT are consistent with those obtained from the standards disk-based IDR measurement method for the M and L size crystals but approximately 50% lower for the S crystal size class. This is probably because crystals in the small size class are being dissolved too fast and are excluded by the sorting algorithm due to their short lifespan (as in Table 1, they may exist in only one time step), and those that pass are significantly retarded by the saturation of the dissolution media. This same reason also partially impacted the measurements for medium- and large-sized crystals. The measured values for medium and large crystals are much closer on average to standard measurement, although these results come from a large range of values. For an illustration of this phenomenon, I_n_DR was plotted spatially distributed in the experiment (Figure 12) from which a group of crystals with a very low dissolution rate can be clearly observed at the edge and at the bottom of the measured sample. In the case of the standard IDR measurement, the whole disc is exposed to a fast-moving dissolution medium, whereas in the pack bed, particles are exposed to fresh dissolution medium sequentially, and the local fluid velocity within the layer is probably not entirely uniform.

**Table 3:** Direct Comparison of the Mean Individual Dissolution Rate (I_n_DR) Evaluated from Microtomography and the Standard Intrinsic Dissolution Rate (IDR) Evaluated by the Disk Method

**crystal size class** (μm)	**I**_**n**_**DR** (mg·cm^–2^·min^–1^)	**IDR** (mg·cm^–2^·min^–1^)
<250 (S)^a^	13.73	26.89
300–400 (M)	24.20	26.97
400–500 (L)	29.03	26.60

a Refers to ranges 180–250 μm for I_n_DR and 25–100 μm for IDR measurements.

On the other hand, when we look at the single-crystal dissolutions, single-crystals can measurably dissolve much faster (2–3 times faster in the obtained maximum) than the value measured by the standard method. This observation means that the standard method is very robust and insensitive to the crystal size. However, it probably does not produce a 100% correct value of dissolution rate as there are factors which retard dissolution even in standard measurement, such as the water flow pattern around the sample in the disc apparatus or the mitigation of individual crystal properties by compressing crystals ahead of the dissolution experiment.

The difference in crystal size classes in Table 3, that is 180–250 μm for the I_n_DR, and 25–100 μm for the IDR measurements differ on purpose: while the class range for the SR-pXRT measurement is inherent to the measurement setup itself, a smaller size class was selected for the IDR measurement. That displays the robustness of the IDR disc setup, showcasing the lack of IDR change with size.

## 4 Conclusions

The presented method based on time-resolved X-ray microtomography can successfully track individual crystals and their shape changes during dissolution. Based on calculated shape factors, individual crystals can be identified at each time step of the dissolution process. The individual dissolution rates for each tracked crystal can then be automatically calculated. Thus, the method provides a level of detail comparable with image analysis of single-crystal dissolution while simultaneously covering a population of at least several tens to hundreds of crystals. The method is therefore complementary to established intrinsic dissolution rate (IDR) measurement by the disk methods, which provides a population-average value without single-particle resolution. The method was demonstrated using NaCl as a model material, chosen thanks to its good contract relative to water. The values of the individual dissolution rate within the crystal population were found to be unexpectedly broad. The observed phenomenon that NaCl crystals tend to dissolve faster as they get smaller may be caused by their shape change in the final stages of dissolution and the specific rate at which the ion pairs leave the crystal lattice.

The presented method comes with not only advantages but also with limitations. It requires a specialized measurement cell and synchrotron source, which means it is probably not suitable for routine measurements. The use of this method could be justified for special use cases such as the measurements of highly valuable pharmaceutical materials with site-specific dissolution rates, or with low-soluble drugs such as those used in long-acting injectable depot systems.^33^ This study also highlighted several important aspects of the experimental design that could be done differently in future experiments. These include the importance of flow distribution uniformity within the measurement cell (possible wall effects) and the importance of the local saturation of the dissolution medium, which controls the local driving force for dissolution. With the results described in this article, those aspects of the dissolution experiment can be resolved e.g. by using the appropriate flow rate of the dissolution medium or by adjusting the concentration of crystals within the packed bed, ensuring a dissolution process unrestricted by solvent saturation.
